# Effects of Dietary GABA Levels on Growth, Muscle Quality, and Liver Lipid Profile: Insights from Lipidomics in Juvenile Yellowfin Seabream *Acanthopagrus latus*

**DOI:** 10.3390/foods14162761

**Published:** 2025-08-08

**Authors:** Guanrong Zhang, Yanjian Yang, Zini Huang, Shishi Zheng, Xinyu Feng, Ju Li, Fang Chen, Yuanyou Li

**Affiliations:** 1 Nansha-South China Agricultural University Fishery Research Institute Guangdong, Guangzhou 511464, China; zhang-gr@139.com (G.Z.); yangyanjian@gznshnyyyjy1.wecom.work (Y.Y.); hznlemon@163.com (Z.H.); lddjul5@163.com (J.L.); 2 College of Marine Sciences, South China Agricultural University, Guangzhou 510642, China; 13635014966@163.com (S.Z.); 18998363146@163.com (X.F.); chenfang@scau.edu.cn (F.C.)

**Keywords:** gamma-aminobutyric acid, *Acanthopagrus latus*, growth performance, muscle quality, lipid profiles

## Abstract

Gamma-aminobutyric acid (GABA), a major inhibitory neurotransmitter, is used as a feed additive in aquaculture. However, its effects on muscle quality and lipid metabolism in fish remain understudied. Therefore, three diets supplemented with 0%, 0.01%, and 0.10% GABA were fed to juvenile *Acanthopagrus latus* (initial weight: 9.96 g) for 9 weeks, followed by analyses of growth performance, muscle quality indices, and hepatic lipid profiles. Fish fed 0.01% GABA showed the highest weight gain rate (*p* < 0.05). Their muscles exhibited improved muscle texture, higher levels of essential/non-essential and flavor amino acids, and a higher proportion of long-chain polyunsaturated fatty acids (LC-PUFAs), including docosahexaenoic acid (DHA) and eicosapentaenoic acid (EPA), along with triglycerides and cardiolipin enriched in LC-PUFA chains (*p* < 0.05). Moreover, their livers demonstrated increased levels of triglycerides, phosphatidylethanolamine, and LC-PUFA, along with reduced levels of phosphatidylcholine, lysophosphatidylcholine, lysophosphatidylethanolamine, and phosphatidylserine (*p* < 0.05). These results suggest that 0.01% GABA supplementation improves growth performance, enhances flesh quality, and optimizes liver lipid profiles in *A. latus*.

## 1. Introduction

Gamma-aminobutyric acid (GABA) is a critical signaling molecule and the primary inhibitory neurotransmitter in the central nervous system. It is a naturally occurring non-proteinogenic amino acid that is widely distributed in animals, plants, and microorganisms [1]. GABA exerts diverse physiological functions including antioxidant activity, blood pressure reduction, anti-aging effects, and enhanced hepatorenal functions [2,3,4,5,6,7,8]. At present, GABA is currently marketed as a food supplement, supported by studies demonstrating its health-promoting benefits for humans [9]. Interestingly, in fish, GABA can also promote growth, regulate appetite, improve feed utilization, enhance stress resistance and immunity, mitigate cell apoptosis, and support gut health [10,11,12,13]. For instance, dietary supplementation with 100 mg/kg GABA improved growth performance, feed intake, and glucose homeostasis in largemouth bass (*Micropterus salmoides*) [11], and 160 mg/kg GABA alleviated oxidative stress, inflammation, apoptosis, and microbiota imbalance in turbot (*Scophthalmus maximus*) [12]. These findings highlight GABA’s potential as a novel, effective, and safe feed additive in aquaculture.

Fish serve as the primary source of long-chain polyunsaturated fatty acids (LC-PUFAs) in the human diet [14]. Studies indicate that the intake of LC-PUFAs is essential for human health [15]. However, these fatty acids are prone to lipid peroxidation, which reduces fish flesh quality [16]. Recent studies have found that exogenous GABA can mitigate lipid peroxidation to improve pork quality [17]. Additionally, studies in mammals and poultry have shown that GABA can regulate hepatic glucose metabolism and lipid metabolism [2,18,19,20,21]. However, the effects of GABA on fish muscle quality and lipid metabolism remain unexplored.

*Acanthopagrus latus*, prized for its tender flesh, delicious taste, and high nutritional value, is both a consumer favorite and a key aquaculture species along China’s southern coast [22,23]. Previous studies indicate that most fish species require dietary GABA within a range of approximately 75 to 527 mg/kg [9,10,11,24,25,26]. Therefore, this study was designed primarily to explore the physiological responses of juvenile *A. latus* to different levels of dietary GABA (0%—control, 0.01%—moderate level, 0.10%—high level), using lipidomics and biochemical analyses to evaluate changes in muscle nutritional composition, lipid profiles, texture characteristics, and hepatic lipid metabolism. These findings will advance our understanding of GABA’s regulatory roles in fish muscle quality and lipid metabolism, thereby contributing to aquatic nutrition science.

## 2. Materials and Methods

The animal experiments were approved by the Institutional Animal Care and Use Committee (IACUC) of South China Agricultural University (approval number: SYXK-2019-0136, approved on 7 June 2022).

### 2.1. Experimental Diets

Three isonitrogenous (50%) and isolipidic (11%) diets were formulated with GABA inclusions of 0% (L-0), 0.01% (L-0.01), and 0.10% (L-0.1). The actual GABA concentrations quantified by HPLC were 64.93, 136.59, and 772.93 mg/kg diet, respectively. All ingredients were purchased from Guangdong Evergreen Feed Industry Co., Ltd. (Zhanjiang, China). The ingredients were ground and thoroughly mixed, and then combined with oil and 35% distilled water, followed by further mixing. The mixture was pelletized into 2.0 mm pellets using a pellet making machine (SLC-45, Fishery Machinery and Instrument Research Institute, Shanghai, China), air-dried, sealed in bags, and stored at –20 °C for future use. The feed formulations used in this experiment are shown in Table 1.

### 2.2. Fish Husbandry

The *A. latus* were purchased from a local hatchery and initially reared in a net cage in a pond located at the Nansha-South China Agricultural University Fishery Research Institute (Guangzhou, China). Then, the fish were fed the base diet for 8 days to acclimate to the experimental conditions. Subsequently, 225 juvenile fish (initial weight: 9.96 g) were arbitrarily allocated into 9 cages (1 m × 1 m × 1.5 m; 25 fish/cage; triplicate groups). The feeding trial lasted for 63 days. The daily dead fish were recorded in order to calculate the survival rate. Throughout both acclimatization and the feeding trial, juveniles were fed to satiation twice daily at 6:00 and 17:30. The feeding trial was conducted using floating pellets, and each cage was surrounded by fine-mesh netting (0.355 mm) to prevent feed from escaping or drifting. During the trial, the salinity ranged from 2 to 5‰, the water temperature ranged from 20.2 to 27.1 °C, the ammonia nitrogen concentration was <0.05 mg/L, and the dissolved oxygen above 6 mg/L.

### 2.3. Sample Collection

At the end of the 63-day feeding trial, all fish underwent a 24 h fasting period before being weighed to determine growth performance metrics. Subsequently, twelve fish from each cage were arbitrarily selected, anesthetized with 0.01% 2-phenoxyethanol, and then sampled. Four fish per cage were sampled to measure muscle texture, four were sampled to measure whole body proximate composition, and the remaining four were sampled to harvest liver (in duplicate) and muscle (in quadruplicate) for the analysis of hepatic lipidome and fatty acid profiles, muscular lipidome, proximate composition, and amino acid and fatty acid profiles. The tissue samples were quickly frozen in liquid nitrogen and stored at −80 °C until analysis.

### 2.4. Analysis of Nutritional Compositions and Texture Parameters

The proximate composition (moisture, crude protein, crude lipid, and ash) of both diets and tissue samples was analyzed according to the AOAC official methods [27]. For the determination of muscular amino acid composition, tryptophan (Try) was quantified using HPLC (Agilent 1260, Santa Clara, CA, USA) after alkaline hydrolysis with a 4.3 M NaOH solution. Cysteine (Cys) and other amino acids were quantified using an amino acid analyzer (Hitachi LA8080, Tokyo, Japan) following oxidative acid hydrolysis with performic acid solution, sodium metabisulfite solution, and 6.8 M HCl, and acid hydrolysis with 6 M HCl, according to the protocol of Lin et al. [28]. The edible quality and texture parameters of the muscle were assessed following the methods described in our previous report [29,30]. Briefly, two dorsal muscle samples were collected from either side of the dorsal fin. One dorsal muscle sample was cut into two pieces for the measurement of cooked meat rate (CMR), while the other muscle was used for texture analysis. The CMR was measured as follows: 1 g of dorsal muscle (W1) was cooked at 100 °C for 5 min, then cooled and weighed again (W2). The CMR was calculated using the following formula: CMR (%) = W2/W1 × 100%.

### 2.5. Analysis of Lipidomic AMD Fatty Acid Profile

For hepatic and muscular lipidomics analysis, fish from each cage were pooled in pairs to form six biological replicates per treatment group. The lipids in tissue were extracted using the chloroform methanol solution (2:1) according to our previously published protocol [31]. The extracted lipids were then analyzed via LC-MS based on the methodology of Li et al. [32]. Raw data were processed for lipid annotation and filtering using LipidSearch software (version 4.1.2; Thermo Fisher Scientific, Waltham, MA, USA), with total peak area normalization applied to each sample. Partial least squares discriminant analysis (PLS-DA) was performed using the ropls R package (version 1.6.2; Vienna, Austria) [33] to evaluate inter-group separations, with statistically significant lipids identified by variable importance in projection (VIP) scores ≥ 1.0 and adjusted *p*-values ≤ 0.05. Differential lipids (top 50 molecules ranked by fold-change) across pairwise comparisons (L-0 vs. L-0.01, L-0 vs. L-0.1, and L-0.01 vs. L-0.1) were visualized through hierarchical clustering analysis using the heatmap package in the R software (v3.3.2; Vienna, Austria) [34]. Fatty acid profiles in hepatic and muscle tissues were determined according to the methodology of Li et al. [35]. Briefly, lipids from the tissues were extracted using the chloroform methanol solution (2:1), and lipid methyl esterification was carried out with 0.5 M KOH-methanol (0.5 M) and 15% boron trifluoride methanol complex solution. The products were separated using a gas chromatograph (Agilent GC-7890B, Santa Clara, CA, USA). The fatty acids were classified and identified based on fatty acid standards (Sigma, Merck KGaA, Darmstadt, Germany) and expressed as a percentage of total fatty acids.

### 2.6. Statistical Analysis

All data are expressed as mean ± standard error of the mean (SEM). After testing for normality and homogeneity, the data were analyzed by one-way ANOVA followed by Duncan’s multiple range test using SPSS 20.0 (IBM Corp., Armonk, NY, USA). Statistical significance was set at *p* < 0.05. The linear and quadratic effects of dietary GABA levels were determined using regression analysis, with dietary GABA levels as the independent variable.

## 3. Results

### 3.1. Growth Performance

There were no differences (*p* > 0.05) in the survival rate (SUR) among the three groups. However, the final weight, weight gain rate (WGR), and specific growth rate (SGR) were significantly higher (*p* < 0.05) in the L-0.01 group compared with the other groups. The feed intake (FI) and feed conversion ratio (FCR) of the L-0.01 group were significantly lower (*p* < 0.05) than those of the L-0 group, but no difference (*p* > 0.05) was observed when compared with the L-0.1 group (Table 2).

### 3.2. Whole Body and Muscle Proximate Composition

The dietary GABA levels did not significantly affect the whole body lipid content or the muscle moisture and protein content (*p* > 0.05; Table 3). However, the L-0.01 group exhibited a significantly higher whole body protein and muscle lipid content (*p* < 0.05), yet a significantly lower muscle ash content (*p* < 0.05) compared with the other treatments. This group also showed a significantly lower whole body moisture and ash content than the L-0 group (*p* < 0.05), but no difference relative to the L-0.1 group (*p* > 0.05).

### 3.3. Muscle Amino Acid Composition

The levels of all muscular amino acids exhibited a quadratic distribution (*p* < 0.05; Table 4). The L-0.01 group displayed significantly elevated levels of Ala, Arg, Asp, Cystine, Glu, Gly, His, Ile, Leu, Lys, Met, Phe, Pro, Ser, Thr, Try, Tyr, and Val, along with total essential (EAAs), non-essential (NEAAs), and flavor amino acids (FAAs) compared with the other groups (*p* < 0.05). While Cys concentration was significantly lower in the L-0.01 group compared with the L-0.01 group (*p* < 0.05), it did not differ significantly from the L-0 group (*p* > 0.05).

### 3.4. Muscle Texture

As shown in Table 5, the muscle hardness, cohesiveness, shear force, and cooked meat rate were not significantly (*p* > 0.05) influenced by dietary GABA levels. However, the muscle springiness, chewiness, and gumminess were significantly higher (*p* < 0.05) in the L-0.01 group compared with the other groups.

### 3.5. Results of Lipidomics Analysis of the Liver and Muscle

**Figure 1:**
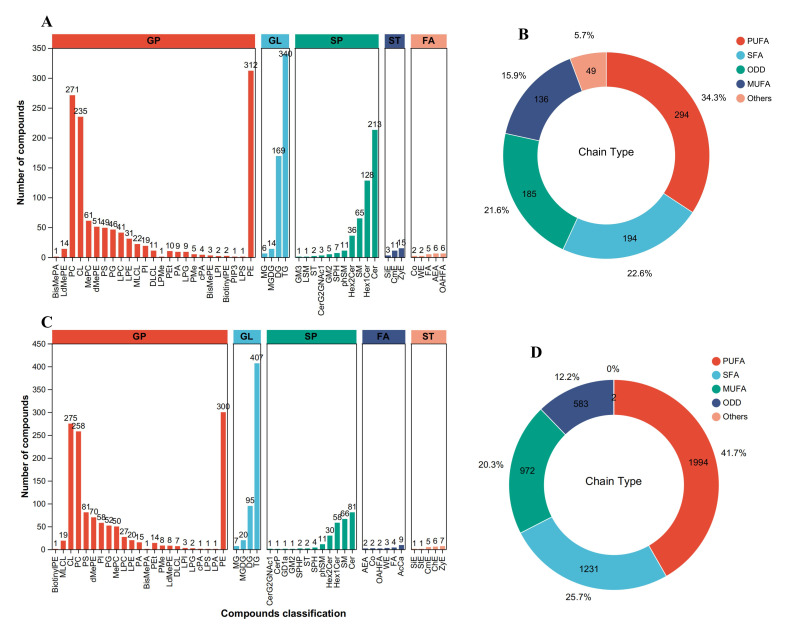
Overall distribution of major lipid subclasses (**A**,**C**) and chain types (**B**,**D**) in the liver (**A**,**B**) and muscle (**C**,**D**) of *Acanthopagrus latus* in all groups. **A**,**C**: the *x*-axis represents the number of lipids identified for each subclass, while the *y*-axis shows the names of the subclasses. FAs, fatty acyls; GLs, glycerolipids; GPs, glycerophospholipids; SPs, sphingolipids; STs, sterol lipids; SFAs, saturated fatty acyls; MUFAs, monounsaturated fatty acyls; PUFAs, polyunsaturated fatty acyls; ODDs, odd-numbered fatty acyls.

**Figure 2:**
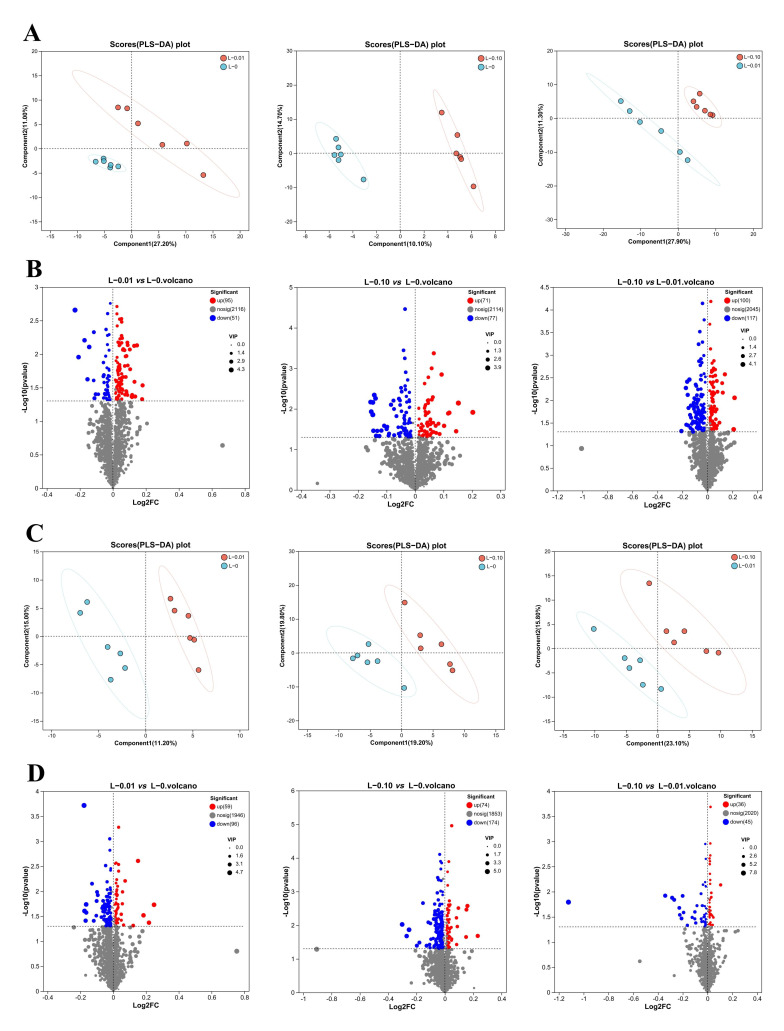
Lipidomics analysis in livers (**A**,**B**) and muscles (**C**,**D**) of *Acanthopagrus latus* fed diets with varying GABA levels. (**A**,**C**): PLS-DA score plot of the L-0 and L-0.01 groups, the L-0 and L-0.1 groups, and the L-0.01 and L-0.1 groups. (**B**,**D**): Volcano plot of L-0.01 vs. L-0, 0.1 vs. L-0, and 0.1 vs. L-0.01, respectively. VIP > 1.0, which was obtained using the PLS-DA model, and *p* < 0.05 were used to screen differential metabolites. In subsequent visualizations: red, green, and gray dots represent upregulated metabolites, downregulated metabolites, and metabolites with no difference, respectively.

**Figure 3:**
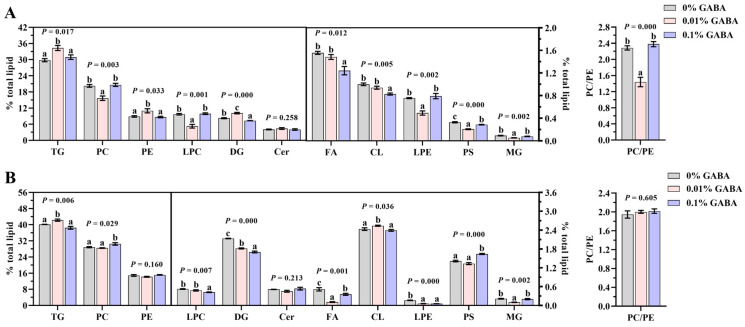
Relative abundance (% of total lipids) of major lipid subclasses in liver (**A**) and muscle (**B**) of *Acanthopagrus latus* fed diets with graded GABA supplementation levels. Values represent means ± SEM (n = 3 replicate treatment group). Within each lipid subclass, bars without shared lowercase letters indicate significant differences (*p* < 0.05).

**Figure 4:**
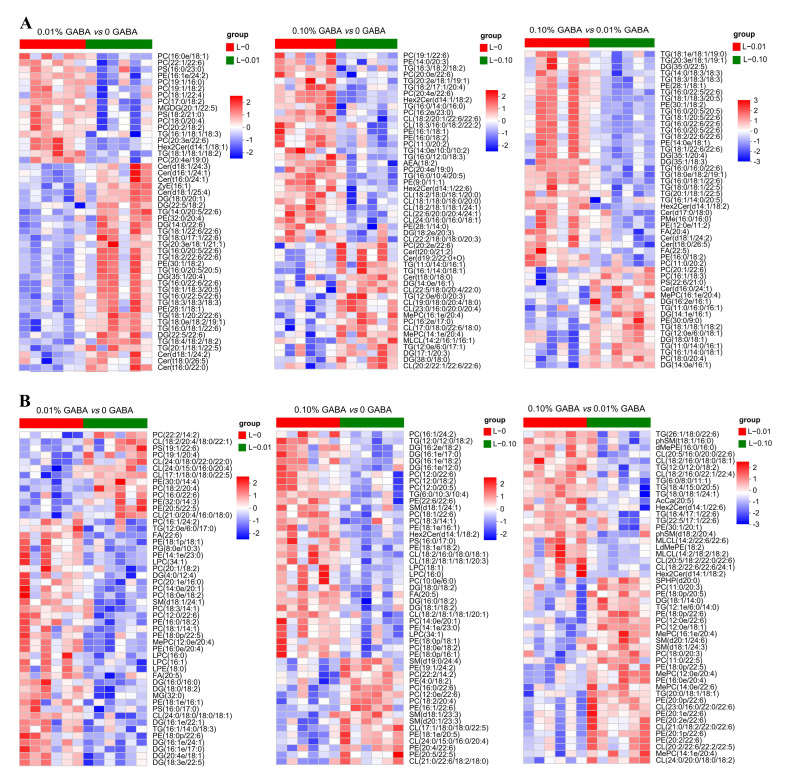
The hierarchical clustering of significantly differential lipid molecules in livers (**A**) and muscles (**B**) of *Acanthopagrus latus* fed diets with varying GABA levels.

The results of the lipidomics analysis of the liver and muscle are shown in Figure 1, Figure 2, Figure 3 and Figure 4. A total of 2262 and 2101 lipid molecules were identified in the liver and muscle, respectively (Figure 1A,C). The most abundant lipids subclasses were triglyceride (TG), phosphatidylethanolamine (PE), phosphatidylcholine (PC), and cardiolipin (CL), with abundances of 15.03%, 13.79%, 11.98%, and 10.39% in the liver, and 19.37%, 14.28%, 12.28%, and 13.09% in the muscle, respectively (Figure 1A,C). Furthermore, the content of polyunsaturated fatty acyls was the highest, comprising 34.3% in the liver and 41.7% in the muscle, respectively (Figure 1B,D).

PLS-DA demonstrated distinct clustering between all treatment groups (L-0 vs. L-0.01, L-0 vs. L-0.1, L-0.01 vs. L-0.1) in both tissues (Figure 2A,C), indicating that GABA supplementation substantially altered lipid metabolism. Volcano plots quantified these changes with 146 (95 upregulated, 51 downregulated), 148 (71 upregulated, 77 downregulated), and 217 (100 upregulated, 117 downregulated) hepatic differential lipids (DLs), and 155 (59 upregulated and 96 downregulated), 248 (74 upregulated and 174 downregulated), and 81 (36 upregulated and 45 downregulated) muscular DLs for respective pairwise comparisons (Figure 2B,D).

Specifically, the L-0.01 group exhibited significantly higher proportions of hepatic TG, PE, and diacylglycerols (DGs), along with elevated muscular TG and CL (*p* < 0.05; Figure 3A,B). Conversely, lower proportions were observed for hepatic PC, lysophosphatidylcholine (LPC), lysophosphatidylethanolamine (LPE), phosphatidylserine (PS), monoacylglycerols (MGs), muscular fatty acids (FAs), and MG, as well as the hepatic PC/PE ratio (*p* < 0.05). When comparing L-0.01 to L-0.1, hepatic FA and CL increased while muscular LPC increased (*p* < 0.05); meanwhile muscular PC and PS decreased (*p* < 0.05). These differences were non-significant relative to L-0 (*p* > 0.05). Muscular LPE was reduced in L-0.01 versus L-0 (*p* < 0.05) but unchanged versus L-0.1. Ceramide (Cer) proportions in both tissues, muscular PE, and muscular PC/PE ratio remained unaffected by GABA (*p* > 0.05).

Furthermore, a cluster analysis using the top 50 lipids was performed to further evaluate the influence of dietary GABA supplementation on the hepatic and muscular lipid profiles (Figure 4A,B). Hepatic PUFA-enriched TG (particularly EPA/DHA species) increased significantly at 136.59 mg/kg GABA (L-0.01) compared with 64.93 mg/kg (L-0) (*p* < 0.05) but decreased at 772.93 mg/kg (L-0.1) (*p* < 0.05). This pattern was inverted for SFA/MUFA-rich PC. In muscle, PUFA-bound PC, PE, and CL (especially EPA/DHA-containing) significantly increased in L-0.01 versus L-0 (*p* < 0.05), while EPA/DHA-enriched TG, PC, PE, and CL decreased significantly in L-0.1 versus L-0.01 (*p* < 0.05).

### 3.6. Fatty Acid Profile of the Liver and Muscle

**Figure 5:**
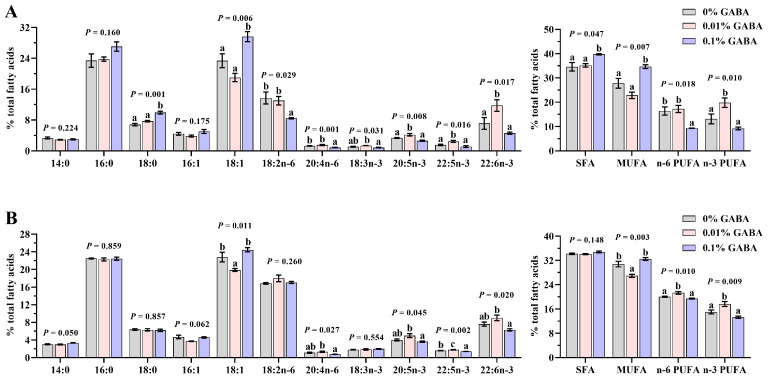
Fatty acid composition in livers (**A**) and muscles (**B**) of *Acanthopagrus latus* fed diets with graded GABA supplementation levels. Values represent means ± SEM (n = 3 replicate treatment group). Within each lipid subclass, bars without shared lowercase letters indicate significant differences (*p* < 0.05). SFAs, saturated fatty acids; MUFAs, monounsaturated fatty acids; PUFAs, polyunsaturated fatty acids.

The L-0.01 group showed significantly elevated hepatic EPA, docosapentaenoic acid (DPA), DHA, and n-3 PUFA, along with increased muscular DPA, n-6 PUFA, and n-3 PUFA, while muscular MUFA decreased relative to other groups (*p* < 0.05; Figure 5). When comparing L-0.01 to L-0.1, hepatic linoleic acid (LA), arachidonic acid (ARA), α-linolenic acid (ALA), and n-6 PUFA increased significantly, as did muscular ARA, EPA, and DHA (*p* < 0.05). Conversely, hepatic 18:0, 18:1, ΣSFA, and MUFA decreased (*p* < 0.05). These differences were non-significant between the L-0.01 and L-0 groups (*p* > 0.05). Dietary GABA did not affect hepatic 14:0, 16:0, or 16:1, nor muscular 14:0, 16:0, 18:0, 16:1, LA, or ALA proportions (*p* > 0.05).

## 4. Discussion

GABA has growth-promoting and antioxidant functions and is used as a feed additive in fish farming [10,12]. In this study, 136.59 mg/kg GABA (supplementing 0.01% in the feed) improved the growth performance of *A. latus*. Similarly, dietary GABA supplementation at 237 mg/kg improved growth performance and feed utilization in olive flounder (*Paralichthys olivaceus*) [25], 527 mg/kg GABA showed similar effects in gibel carp (*Carassius auratus gibelio*) [10], and 200–500 mg/kg GABA enhanced these parameters in Nile tilapia (*Oreochromis niloticus*) [36]. The growth-promoting effect of GABA may be related to its ability to enhance feed utilization and reduce stress in cultured fish [37]. In this study, 136.59 mg/kg GABA significantly reduced the FCR, further proving that GABA can improve feed utilization. However, in this study, high levels of GABA (772.93 mg/kg) inhibited the growth of *A. latus*, partly due to excess GABA affecting cellular calcium ion levels, which in turn suppresses the release of growth hormones, though additional causes and mechanisms still require further investigation [10]. Similar phenomenon has also been observed in species like whiteleg shrimp (*Litopenaeus vannamei*), Jian carp (*Cyprinus carpio*), olive flounder, and Nile tilapia [25,26,38,39].

Fish growth is closely related to protein deposition [40]. This study further demonstrated a positive correlation between fish growth and whole body protein content, with the highest protein levels observed in the group exhibiting the best growth (136.59 mg/kg GABA group). Furthermore, 136.59 mg/kg GABA increased the muscular EAA, NEAA, and FAA levels in this study. Similar findings have been observed in piglets, where GABA supplementation increases the levels of most naturally occurring amino acids in the intestinal mucosa, although the underlying regulatory mechanisms remain unclear [41]. The quality of fish flesh is closely associated with the muscular EAA and FAA levels [42]. A higher muscular EAA content generally indicates greater nutritional value, while FAA levels impact meat freshness [42]. Our results indicate that dietary GABA at 136.59 mg/kg promotes the accumulation of EAA and FAA in muscle, enhancing the fish’s nutritional value.

Interestingly, the 136.59 mg/kg GABA increased muscle lipid content. Similarly, the optimum dietary GABA level can increase the muscle lipid content in whiteleg shrimp [43]. This may be linked to the regulatory effects of GABA on energy metabolism [43]. Furthermore, further analysis of the muscle lipidome revealed that the muscle of *A. latus* contained a total of 2101 lipid species, which was significantly higher than in China’s domestic pork (1180 lipid species), Taihe black-boned silky fowl (1127 lipid species), and donkey (1146 lipid species) [32,44,45]. Additionally, TGs were the predominant lipid subclasses in the muscle of *A. latus*, followed by PC and PE, with the three classes accounting for approximately 84% of the total lipids, which is consistent with findings in tilapia muscle [46]. In this study, 136.59 mg/kg GABA significantly increased the muscle TG and CL content, indicating that dietary GABA levels influence muscle lipid profiles. Moreover, appropriate GABA levels enhanced the content of PC, PE, and CL enriched in PUFA chains, particularly EPA and DHA, while excessive GABA reduced the content of TG, PC, PE, and CL enriched with EPA and DHA. This indicates that optimal GABA levels (specifically 136.59 mg/kg) enhance the deposition of PUFA, particularly EPA and DHA, in muscle. The significantly higher proportions of ARA, EPA, DPA, and DHA observed in this group’s muscle tissue directly corroborate this finding. Although PUFA is inherently prone to lipid peroxidation [16], the observed accumulation may be attributed to GABA’s antioxidant capacity. GABA has been demonstrated to mitigate lipid peroxidation in meat (e.g., pork), improving quality [17], suggesting that enhanced antioxidant protection likely facilitates PUFA deposition in fish muscle. Additionally, GABA may function as a multifunctional carbon–nitrogen reservoir that promotes the deposition of unsaturated fatty acids [47]. For human health, the intake of appropriate levels of ARA, EPA, and DHA is very important and essential [15]. The higher proportion of these fatty acids indicates greater nutritional value. The results suggested that dietary GABA at 136.59 mg/kg promotes the deposition of LC-PUFA, particularly DHA and EPA, in muscle.

In fish, muscle lipid is one of the main factors affecting meat quality and is positively correlated with gumminess [29]. A moderate increase in muscle lipid levels has been reported to improve the tenderness and palatability of the flesh [48]. In species such as gibel carp and golden pompano (*Trachinotus ovatus*), an elevated intramuscular lipid content has been associated with improved meat quality [29,48]. Additionally, muscle texture parameters, such as chewiness, springiness, and gumminess, etc., are crucial traits for assessing fish flesh quality [40,49]. Generally, fish muscle with higher springiness and chewiness is preferred by consumers [50]. Enhancements in hardness, springiness, and chewiness have been associated with improved flesh quality in species such as largemouth bass and large yellow croaker [51,52]. In our study, fish fed a diet containing 136.59 mg/kg of GABA exhibited greater muscle springiness, chewiness, and gumminess compared with those fed other diets, suggesting that dietary GABA at 136.59 mg/kg can enhance muscle texture, thereby improving the flesh quality of *A. latus.*

As the primary metabolic organ in vertebrates, the liver regulates animal growth through its metabolic functions [53]. Lipids serve essential roles in membrane structure, energy storage, and cellular signaling, while also reflecting metabolic states [54,55]. To assess dietary GABA’s impact on hepatic lipid metabolism, we conducted a comprehensive liver lipidome analysis. A total of 2262 lipid species were identified in the liver of *A. latus*. This was significantly lower than that in the golden pompano (3458 lipid species), but higher than in Dezhou donkeys (1134 lipid species) [31,32]. Similarly to muscle, the major lipids in the liver of *A. latus* are TG, PC, and PE, which account for about 60% of the total lipids, but this is much lower than in its muscle (84%). In the liver of *T. ovatus*, TG, PC, and PE constitute approximately 71.55% of total lipids [31]. Despite marked variation in lipid profiles across species and tissues, TG, PC, and PE consistently represent the primary lipid classes.

Increasing evidence suggests that TG accumulation may serve as a protective mechanism against lipotoxic lipids rather than inducing hepatic damage [56]. Our findings revealed that 136.59 mg/kg GABA elevated hepatic TG, potentially indicating a self-protection response during cell growth. However, in mammals and livestock, GABA can downregulate lipogenesis and enhance lipid degradation, reducing liver TG [18,19,20], consistent with our observed TG reduction at high GABA levels. As the sole phospholipid regulating lipoprotein assembly/secretion, PC critically maintains liver function—its deficiency triggers triglyceride accumulation [57,58]. PE, the second most abundant cellular phospholipid and a key component of the inner mitochondrial membrane, not only maintains membrane structure but also regulates mitochondrial morphology, curvature, and fusion [59]. Altered PC/PE ratios correlate with pathological conditions [60]. Additionally, the accumulation of certain intermediate lipids like LPC can lead to mitochondrial dysfunction and inflammation [61,62]. A study reported that high-fat diets increase the levels of PC, PS, LPC, and the PC/PE ratio, while reducing PE, inducing hepatic steatosis in mice [58]. In this study, 136.59 mg/kg GABA significantly increased PE level, reduced PC, LPC, LPE, and PS levels, and decreased the PC/PE ratio in the liver, suggesting that appropriate GABA levels can regulate liver lipid composition and reduce harmful lipids. Conversely, high dietary GABA level reversed these effects, revealing a nonlinear dose–response relationship, but the underlying mechanisms remain unclear and require further investigation. Furthermore, cardiolipin (CL), known as the “signature phospholipid” of mitochondria, plays a central role in energy production [63]. High dietary GABA levels reduced the CL level of *A. latus*, indicating that high GABA may impair liver energy metabolism. Interestingly, appropriately dosed dietary GABA elevated hepatic TG enriched in PUFA chains—notably EPA and DHA—while high concentrations significantly reduced these lipids, contrasting with PC containing SFA/MUFA chains which exhibited an inverse response. Concurrently, EPA, DPA, DHA, and n-3 PUFA levels also peaked at intermediate GABA doses before declining. Previous studies suggest that appropriate GABA can regulate carbon–nitrogen metabolism and alleviate lipid peroxidation in the hepatopancreas of fish, promoting the deposition of unsaturated fatty acids [64]. However, the reasons for the negative effect of high GABA on PUFA deposition need further investigation. Overall, dietary GABA at 136.59 mg/kg can regulate liver lipid profile, reduce harmful lipid molecules, and promote the deposition of n-3 PUFAs such as EPA, DPA, and DHA, improving lipid metabolism. However, excessive GABA may also lead to an increase in harmful lipid molecules and potentially impair liver energy metabolism in *A. latus*.

## 5. Conclusions

In conclusion, 136.59 mg/kg GABA significantly enhanced the growth performance of *A. latus*. It improved the nutritional value of the muscle by increasing the deposition of EAAs, FAAs, and PUFAs, particularly EPA, DPA, DHA, including PUFA-enriched TG and CL, while also improving muscle texture. Furthermore, it optimized liver lipid profiles by reducing harmful lipids and promoting the deposition of n-3 PUFAs (EPA, DPA, DHA), thereby improving lipid metabolism. To our knowledge, this work provides the first evidence of GABA’s role in modulating fish muscle quality, offering novel insights into its physiological functions in aquaculture nutrition. However, further studies are needed to explore a broader range of dietary GABA levels and to determine the optimal dose across different growth stages, including in larger fish.

## Figures and Tables

**Table 1:** Ingredients and proximate composition of the experimental diets (%, dry matter basis).

	Diets		
Ingredients	L-0	L-0.01	L-0.1
Fish meal	30.00	30.00	30.00
Chicken meal	10.40	10.40	10.40
Soybean meal	20.50	20.50	20.50
Ethanol clostridium protein	5.00	5.00	5.00
Black soldier fly powder	5.00	5.00	5.00
Fish oil	2.00	2.00	2.00
Soybean oil	2.00	2.00	2.00
Lecithin	2.00	2.00	2.00
Cassava starch	10.00	10.00	10.00
α-starch	8.30	8.29	8.20
Vitamin premix ^1^	1.00	1.00	1.00
Mineral premix ^2^	1.00	1.00	1.00
Choline chloride	0.50	0.50	0.50
Monocalcium phosphate	1.50	1.50	1.50
Taurine	0.80	0.80	0.80
GABA	0	0.01	0.10
Proximate composition			
Moisture	7.13	6.67	6.85
Crude protein	50.30	50.46	50.46
Crude lipid	11.04	10.77	10.94
Ash	8.98	8.88	8.52
GABA (mg/kg)	64.93	136.59	772.93

Note: ^1^ Vitamin premix (per kg): VA, 1,100,000 IU; VB12, 8 mg; VB1, 1500 mg; VB2, 2800 mg; D3, 320,000 IU; VK3, 1000 mg; alpha-tocopherol, 8000 mg; acetatecalcium pantothenate, 2000 mg; folic acid, 400 mg; l-ascorbyl-2-polyphosphate, 17,000 mg; nicotinamide, 7800 mg; inositol, 12,800 mg; VB6, 1000 mg. ^2^ Mineral premix (per kg): copper sulfate, 10 mg; cobalt chloride (1%), 50 mg; common salt, 100 mg; ferric sulfate, 80 mg; manganese sulfate, 60 mg; magnesium sulfate, 1200 mg; potassium iodide, 0.8 mg; sodium fluoride, 2 mg; zinc sulfate, 50 mg; zeolite powder, 15.45 g.

**Table 2:** Growth performance and feed utilization of *Acanthopagrus latus* fed diets with different GABA levels.

Items	Diets			Pooled SEM	*p*-Value	Regression (*p*, r^2^)			
	L-0	L-0.01	L-0.1			Linear		Quadratic	
Initial weight (g)	9.82	9.91	10.15	0.146	0.693	0.379	0.112	0.693	0.115
Final weight (g)	28.60 ^a^	31.57 ^b^	27.74 ^a^	0.653	0.009	0.138	0.286	0.009	0.789
WGR (%)	191.53 ^b^	218.86 ^c^	173.27 ^a^	7.002	0.001	0.034	0.497	0.001	0.895
SGR (%/day)	1.70 ^b^	1.84 ^c^	1.60 ^a^	0.037	0.001	0.029	0.517	0.001	0.899
FI, (g/fish)	18.43 ^b^	14.35 ^a^	13.91 ^a^	0.794	0.006	0.098	0.343	0.006	0.817
FCR	1.24 ^b^	0.97 ^a^	1.00 ^a^	0.050	0.018	0.247	0.186	0.018	0.739
SUR (%)	89.33	94.67	92.00	1.886	0.579	0.924	0.001	0.579	0.167

Note: Data represent means from triplicate treatments (n = 3). Within each parameter row, means without a common superscript letter indicate significant differences (*p* < 0.05). WGR, weight gain rate (%) = 100 × (final weight—initial weight)/(initial body weight). SGR, specific growth rate (% day-1) = 100 × [Ln(final weight)—Ln(initial weight)]/days. FI, feed intake (g/fish) = dry diet fed/fish number. FCR, feed conversion ratio = (total dry weight of feed fed)/(final weight—initial weight). SUR, survival rate (%) = 100 × survived fish number/total fish number.

**Table 3:** Whole body and muscle proximate composition of *Acanthopagrus latus* fed diets with different GABA levels.

Items	Diets			Pooled SEM	*p*-Value	Regression (*p*, r^2^)			
	L-0	L-0.01	L-0.1			Linear		Quadratic	
Whole body (%)									
Moisture	73.15 ^b^	70.82 ^a^	71.83 ^ab^	0.426	0.042	0.520	0.061	0.042	0.653
Protein	16.98 ^a^	17.53 ^b^	16.68 ^a^	0.133	0.002	0.051	0.442	0.002	0.869
Lipid	5.22	6.30	5.51	0.311	0.389	0.820	0008	0.389	0.270
Ash	5.81 ^b^	5.16 ^a^	5.43 ^ab^	0.115	0.035	0.687	0.025	0.035	0.673
Muscle tissue (%)									
Moisture	77.87	77.36	77.79	0.115	0.144	0.615	0.038	0.144	0.476
Protein	18.58	18.65	18.48	0.047	0.407	0.238	0.192	0.407	0.259
Lipid	1.76 ^b^	2.80 ^c^	0.97 ^a^	0.285	0.003	0.031	0.509	0.003	0.861
Ash	1.27 ^b^	0.94 ^a^	1.31 ^b^	0.065	0.010	0.229	0.199	0.010	0.787

Note: Data represent means from triplicate treatments (n = 3). Within each parameter row, means without a common superscript letter indicate significant differences (*p* < 0.05).

**Table 4:** Amino acid composition in muscles of *Acanthopagrus latus* fed diets with different GABA levels (g/100 g muscle, dry weight).

Items	Diets			Pooled SEM	*p*-Value	Regression (*p*, r^2^)			
	L-0	L-0.01	L-0.1			Linear		Quadratic	
Ala	4.00 ^a^	4.37 ^c^	4.24 ^b^	0.054	0.000	0.507	0.065	0.000	0.999
Arg	3.98 ^a^	4.33 ^c^	4.18 ^b^	0.050	0.000	0.644	0.032	0.000	0.999
Asp	6.57 ^a^	7.40 ^c^	7.07 ^b^	0.120	0.000	0.594	0.043	0.000	1.000
Cys	0.02 ^a^	0.01 ^a^	0.02 ^b^	0.001	0.001	0.002	0.780	0.001	0.890
Cystine	0.34 ^a^	0.40 ^b^	0.33 ^a^	0.012	0.014	0.171	0.250	0.014	0.757
Glu	8.70 ^a^	9.88 ^c^	9.46 ^b^	0.172	0.000	0.513	0.063	0.000	1.000
Gly	3.49 ^a^	3.66 ^c^	3.58 ^b^	0.025	0.000	0.706	0.022	0.000	0.988
His	1.54 ^a^	1.66 ^c^	1.61 ^b^	0.018	0.001	0.594	0.043	0.001	0.899
Ile	3.00 ^a^	3.31 ^c^	3.11 ^b^	0.045	0.000	0.859	0.005	0.000	0.994
Leu	5.38 ^a^	5.96 ^c^	5.70 ^b^	0.085	0.000	0.699	0.023	0.000	0.999
Lys	6.42 ^a^	7.22 ^c^	6.84 ^b^	0115	0.000	0.762	0.014	0.000	0.999
Met	1.87 ^a^	2.19 ^c^	2.02 ^b^	0.046	0.000	0.911	0.014	0.000	1.000
Phe	3.25 ^a^	3.57 ^c^	3.43 ^b^	0.047	0.000	0.666	0.028	0.000	0.995
Pro	2.36 ^a^	2.56 ^c^	2.44 ^b^	0.029	0.000	0.992	0.000	0.000	0.974
Ser	2.22 ^a^	2.40 ^b^	2.43 ^c^	0.033	0.000	0.049	0.446	0.000	0.998
Thr	2.90 ^a^	3.29 ^c^	3.20 ^b^	0.044	0.000	0.399	0.104	0.000	1.000
Try	0.81 ^b^	0.83 ^c^	0.80 ^a^	0.004	0.000	0.068	0.398	0.000	0.984
Tyr	2.19 ^a^	2.41 ^c^	2.33 ^b^	0.032	0.000	0.537	0.057	0.000	0.991
Val	3.36 ^a^	3.66 ^c^	3.47 ^b^	0.045	0.000	0.883	0.003	0.000	0.999
EAA	32.60 ^a^	36.02 ^c^	34.37 ^b^	0.494	0.000	0.775	0.012	0.000	0.999
NEAA	29.89 ^a^	33.08 ^c^	31.89 ^b^	0.465	0.000	0.542	0.055	0.000	1.000
FAA	22.76 ^a^	25.30 ^c^	24.34 ^b^	0.370	0.000	0.550	0.053	0.000	1.000

Note: Data represent means from triplicate treatments (n = 3). Within each parameter row, means without a common superscript letter indicate significant differences (*p* < 0.05). EAAs: essential amino acids are the sum of Arg, His, Ile, Leu, Lys, Met, Phe, Thr, Try, and Val. NEAAs: non-essential amino acids are the sum of Ala, Asp, Cys, Cystine, Glu, Gly, Pro, Ser, and Tyr. FAAs: flavor amino acids are the sum of Asp, Glu, Gly, and Ala.

**Table 5:** Texture parameters and cooked meat rate of muscle of *Acanthopagrus latus* fed diets with different GABA levels.

Items	Diets			Pooled SEM	*p*-Value	Regression (*p*, r^2^)			
	L-0	L-0.01	L-0.1			Linear		Quadratic	
Hardness (gf)	96.31	108.30	107.59	2.807	0.146	0.319	0.099	0.146	0.348
Springiness (mm)	0.47 ^a^	0.54 ^c^	0.52 ^b^	0.010	0.000	0.354	0.086	0.000	0.871
Chewiness (mJ)	24.61 ^a^	33.78 ^b^	24.98 ^a^	1.427	0.001	0.272	0.119	0.001	0.801
Gumminess (mJ)	49.45 ^a^	58.42 ^c^	52.72 ^b^	1.338	0.000	0.876	0.004	0.000	0.958
Cohesiveness	0.56	0.56	0.55	0.004	0.555	0.319	0.099	0.555	0.123
Shear force (gf)	589.26	602.76	586.75	21.34	0.956	0.867	0.003	0.956	0.010
Cooked meat rate (%)	89.99	87.83	88.98	0.705	0.452	0.902	0.002	0.452	0.162

Note: Data represent means from triplicate treatments (n = 3). Within each parameter row, means without a common superscript letter indicate significant differences (*p* < 0.05).

## Data Availability

The original contributions presented in the study are included in the article, further inquiries can be directed to the corresponding author.

## Abbreviations

The following abbreviations are used in this manuscript:

ALA	linolenic acid
ARA	arachidonic acid
Cer	ceramide
CL	cardiolipin
CMR	cooked meat rate
DHA	docosahexaenoic acid
DGs	diacylglycerols
DPA	docosapentaenoic acid
EAAs	essential amino acids
EPA	eicosapentaenoic acid
FAs	fatty acids
FAAs	flavor amino acids
FCR	feed conversion ratio
FI	feed intake
GABA	gamma-aminobutyric acid
HCl	hydrochloric acid
HPLC	high performance liquid chromatography
LA	linoleic acid
LC-MS	liquid chromatography–mass spectrometry
LPC	lysophosphatidylcholine
LPE	lysophosphatidylethanolamine
MGs	monoacylglycerols
MUFAs	monounsaturated fatty acids
NEAA	non-essential amino acid
PC	phosphatidylcholine
PE	phosphatidylethanolamine
PLS-DA	partial least squares discriminant analysis
PUFAs	polyunsaturated fatty acids
PS	phosphatidylserine
SEM	standard error of the mean
SFAs	saturated fatty acids
SUR	survival rate
SGR	specific growth rate
TG	triglyceride
VIP	variable importance in projection
WGR	weight gain rate
